# The Effect of Perinatal Education on Iranian Mothers’ Stress and Labor Pain

**DOI:** 10.5539/gjhs.v6n1p61

**Published:** 2013-10-14

**Authors:** Mozhgan Firouzbakht, Maryam Nikpour, Hajar Salmalian, Farideh Mohsenzadeh Ledari, Sorya Khafri

**Affiliations:** 1 Department of Midwifery, Babol Branch, Islamic Azad University, Babol, Iran; 2 Department of Obstetrics and Gynecology, Babol University of Medical Sciences, Babol, Iran; 3 Fatemezahra Infertility and Reproductive Health Research Center, Department of Midwifery, Babol University of Medical Sciences, Babol, Iran

**Keywords:** perinatal, education Iran, labor pain

## Abstract

Lack of sufficient knowledge about the unknowns of pregnancy increases stress and requires more medical interventions. This study was conducted to assess the effects of prenatal education on mothers’ stress and labor. This clinical trial was conducted to study 195 women (132 in the control group and 63 in the experimental group) who had attended healthcare centers in the city of Amol after their 16th gestational week. The experimental group participated in educational classes to learn how to experience a safe childbirth for 6-8 sessions of 1.5 hours almost every three weeks. The control group received only a routine care, pain assessment scales like Visual Analogue Scale (VAS) and McGill questionnaire, and Hospital Anxiety and Depression Scale (HADS) were employed to collect data. The data were analyzed using SPSS software through t-test and Chi Square test to compare the groups. The results of the t-test showed a meaningful difference in levels of stress felt by the experimental group compared to control group (p=0.002). The Visual Analogue Scale suggested that in the transitional stages (8-10 cm cervical dilation), the level of pain felt by the experimental group was meaningfully lower than that felt by the control group (p=0.03). However, this was not significantly different between the two groups at 3-4 cm cervical dilation and the second stage of childbirth. The McGill scale’s results for measuring pain levels, proved a meaningful difference between the experimental group and the control group (p=0.018). Educational and supportive interventions increased mothers’ knowledge during pregnancy and reduced their fear of unknown environment and people. These trained women learned how to effectively overcome their problems and labor pain.

## 1. Introduction

Labor is one of the most important phenomena and perhaps one of the most painful and stressful experiences that a mother is exposed to in her lifetime (Simkin & Ohara, 2002). More than 90% of the tension and stress of the pregnancy period is related to childbirth (Hosseininasab & Taghavi, 2010). Labor pain is the result of the interactions made by a series of physiologic factors like uterus contractions or cervical dilation (Abushaikha & Oweis, 2005) and psychological factors like stress, anxiety and fear (Abushaikha & Oweis, 2005; Lugina, Mlay, & Smith, 2004; Lang, Sorrell, Rodger, & Lebeck, 2006). Labor pain affects a woman’s emotional control and it can be associated with fear that leads to a prolonged childbirth process and consequently request of mother for an unnecessary cesarean section (Lang et al., 2006). The intense and prolonged uncontrolled labor pain can cause long-term excitement imbalance and can psychologically disturb mother’s health. Moreover, pain and negative excitements can have negative effects on mother-child relationship in the first days of life that are important and vital (Gunasheela & Biliangady, 2004). Mothers and care providers always demand for the guidelines to decrease labor pain or they are without labor (Hosseininasab & Taghavi, 2010). One of the midwives’ goals is preparing good condition for a low risk childbirth and minimum labor for the parturient (Ajh, Sabet Ghadam, & Yonesian, 2011). Today, most countries employ pain relief measures to facilitate childbirth process in that 90% of the women in European countries and 81% of American women are using medical and non-medical measures (Gunasheela & Biliangady, 2004). Mothers’ knowledge of such matters and their active decision in a safe childbirth process is important (Hosseininasab & Taghavi, 2010). It is every pregnant woman’s natural right to get informed of the procedure of natural childbirth and what is going to happen, so they are more prepared to welcome birth or choose their preferred delivery type (Ajh et al., 2011). Perinatal training is one of the strategies that the Ministry of Health has adopted to lessen unnecessary cesarean section and increase natural delivery by reducing mothers’ demand for caesarean section by improving the knowledge of pregnant mothers (Hosseininasab & Taghavi, 2010). Perinatal education is a dynamic process through which parents are informed of the physical and psychological changes during pregnancy, childbirth, becoming a parent and they develop skills to overcome labor and supportive techniques in it (Lee & Holroyd, 2009; Lowdermilk & Perry, 2007). Such education will develop maternal knowledge during pregnancy, childbirth (Koehn, 2008; Malata, Hauck, Monterosso, & McCaul, 2007) and childcare as well it reduces medication during labor and childbirth, pain and stress and improve maternal comfort during childbirth, and obtaining skills to tackle with labor pain by means of physical preparation like relaxation and respiration methods that all will help her to experience a pleasant childbirth (Lowdermilk & Perry, 2007) and increased the probability of their having normal childbirth (Hajian, Shariati, Najmabadi, Yunesian, & Ajami, 2012). Given that mothers need to education during pregnancy and the current policy of the local healthcare system that aims at facilitating the process of childbirth and doing natural delivery more common at hospitals, this study aimed to evaluate the effects of the educational classes on stress and pain level in labor felt by the women admitted to healthcare centers of Amol.

## 2. Materials and Methods

### 2.1 Study Design

This intervention study was conducted from June 2011 to April 2012 on 195 pregnant women at the healthcare center of Amol were studied. The inclusion criteria were as follows: minimum educational qualification for mothers was 5^th^ grade of elementary school, current gestational age of 16 to 20 weeks, mothers’ age of 17 to 35 years old, no contraindication for natural childbirth, lack of any common pregnancy complications and history of medical problems, and not addicted to any kind of drug. Those interested in attending preparation classes for childbirth and were eligible to enter the study population were selected as the experimental group (63 people), participated in 8 sessions of educational class run by four doulas (women having the experience of childbirth and trained in this regard) at two consultation and healthcare centers of Amol. Each session lasted 90 minutes and was divided into 3 parts. Part one was about physiological and anatomical changes during pregnancy, nutritional requirement, psychological health, warning symptoms during pregnancy, the pros and cons of vaginal and caesarean childbirth, different stages of natural delivery, postnatal health, breastfeeding and family planning (theoretical training was presented by means of audiovisual instruments like videos of childbirth). Part two included consultations for 15 minutes in forms of questions and answers. Part three covered neuromuscular training, instruction of proper positions during labor and childbirth, manner of accurate breathing during pregnancy, labor and childbirth, and 30 minutes of relaxation were practiced by pregnant women.

### 2.2 Measurement Instrument

The questionnaires used in this study included demographic information, pregnancy information, and participation information in preparation classes for childbirth. Anxiety and Depression Scale (HADS) was used to assess anxiety, Visual Analogue Scale and McGill questionnaire were used to measure pain. The Persian version of HADS is a standardized tool (Mayou, Springings, Birkhead, & Price 2002). Whose reliability and validity was confirmed by Ali Montazeri et al. (2003). The HADS contains seven questions of four-point Likert scale, where lower scores show less anxiety. Visual Analogue Scale of pain is one of the numerical visual scales with scores from zero to one hundred. Zero means no pain and one hundred shows the highest amount of pain felt by a patient. The VAS had been used in previous studies and shown to be an accurate and sensitive tool for the assessment of the levels of pain in labour (Ip, Chan, & Chien, 2005; Ip, Tang, & Goggins, 2009). The McGill questionnaire is a strong tool for measuring pain levels (Melzack, 1975; Melzack, Taenzer, Feldman, & Kinch, 1981) and its validity and reliability is confirmed in several studies (Brown, Compblell, & Kurtz, 1989; Waters & Raisler, 2003). It contains three dimensions of sensory, emotional and evaluation. In the present study, the emotional aspects of pain has four components including tiring, Sickening, Fearful, being punished by labor pain that were assessed (Melzack, 1975; Melzack et al., 1981). In this questionnaire lower scores indicate less pain. Convenient sampling was used and the sample size was determined through a pilot study.

### 2.3. Data Collection and Intereventions

After the onset of labor pain and hospitalization in experimental group, one of the researchers accompanied the parturient to the labor room and performed all the educated procedures about labor for parturient. These procedures consist of comforting, reassuring, encouraging, talking, and massaging her back, shoulders, and limbs, helping with the best position for different stages of labor, using acupressure to reduce pain, using birth balls, and applying counter-pressure on sacrum, and walking. The midwife completed HADS questionnaire on arrival, VAS at dilation stage 3-4, dilation stage 8-10 and the second stage of childbirth, and by McGill scale tool during the active phase of birth. The control group did not receive any interventions and received the routine care. At delivery room, a trained midwife completed the relevant papers just like for the intervention group, but the patient was controlled by the delivery room personnel. However, because a number of the Imam Ali Hospital personnel had passed preparation classes for childbirth, they applied physiological deep breathing technique and change of position during labor for mothers in the control group.

Finally, the data were entered to SPSS16 software and statistical analysis χ^2^ and t-test were used to analyze the results. The significance level of the test was set at 0.05.

### 2.4 Ethics Review Committee Approval

This study was approved by the Islamic Azad University of the city of Babol for ethics in medical research. Written informed consent was obtained from all participants in the study.

## 3. Results

**Figure 1:**
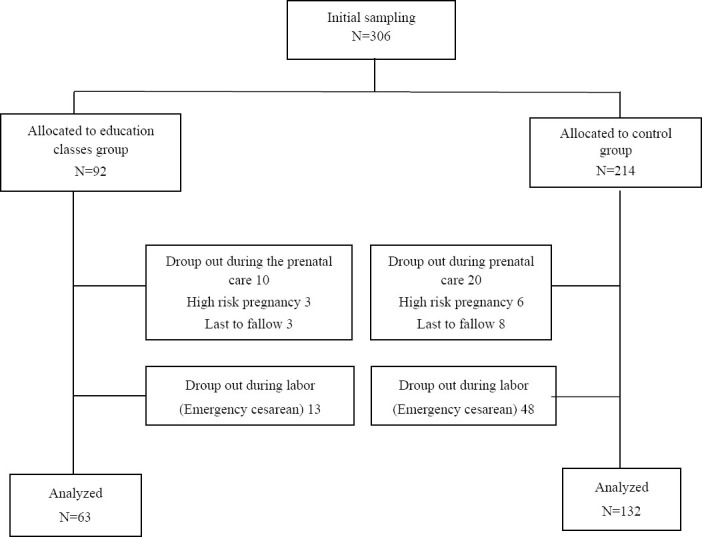
Flow diagram of study

First, 306 cases were enrolled, but 111 people were excluded from the study (Figure 1). Statistical analyses were conducted on 195 cases (132 in the control and 63 in the experimental group). Their mean age was 25.6±5.2. Both groups were matched for mothers’ and their husbands’ occupation, husbands’ education, parity, level of income, and place of residence. Appearance characteristics of mothers such as height (with the mean of 160 cm) and weight (with the mean of 68 kg) were also matched in both groups. There was statistically a meaningful difference between these two groups in terms of the mothers’ education (p=0.003). Fifteen percent of mothers in the experimental group and 50% of mothers in the control group had elementary education (Table 1).

**Table 1:** Demographic characteristics of study groups

Demographic & obstetric characteristics	Classes education group	Control group	P value
Age (mean± SD)	25.42±4.9	25.62±5.28	0.99
Weight (Kg)	68.2±11.8	67.8±13.4	0.88
Length (M)	1.62±0.14	1.59±0.17	0.56
Mother Education			0.003*
Primary	15(24.2%)	66(50.0%)	
High school	28(45.2%)	40(30.3%)	
University	19(30.6%)	26(19.7%)	
Father Education			0.12
Primary	19(31.7%)	71(53.8%)	
High school	20(33.3%)	35(26.5%)	
University	21(35.5%)	26(19.7%)	
Mother Occupation			0.44
Housewife	52(83.9%)	116(87.9%)	
At work	10(16.1%)	16(12.1%)	
Father Occupation			0.08*
employer	17(27.4%)	18(13.6%)	
worker	11(17.7%)	30(22.7%)	
business	34(54.8%)	84(63.6%)	
living space			0.06
City	36(63.2%)	59(48.4%)	
Village	56(90.3%)	63(51.6%)	
Income level			0.72
Low	6(9.7%)	15(11.4%)	
Average	56(90.3%)	117(88.6%)	
Exercise			0.004*
Yes	26(41.9%)	29(22%)	
No	36(58.1%)	103(78%)	

NS: non-significant

The results showed that the mean of stress level felt by the mothers who had been trained was meaningfully less than that in the control group (Table 3).

**Table 2:** Comparison of pain intensity with VAS pain scale in the different phases of labor in two studied groups

Variable	Classes education group(mean± SD)	Control group(mean± SD)	P value
Pain Intensity in the 3-4(cm) Cervical Dilatation	38.13±28.007	40.61±29.56	p=0.58
Pain Intensity in the 8-10(cm) Cervical Dilataion	85.68±18.5	90.99±14.72	*p=0.03
Pain Intensity in the second phase labor	86.08±18.37	90.44±16.64	p=0. 19

**Table 3:** Comparisons Anxiety hospital and pain intensity with McGill pain scale in two studied groups

Variable	Classes education group(mean± SD)	Control group(mean± SD)	P value
Anxiety hospital	14.47±4.69	16.79±4.86	*p=0.002
Tiring	2.45±0.64	2.46±0.62	p=0.95
Sickening	2.16±0.73	2.22±0.66	p=0.56
Fearful	2 ±0.75	2.35±0.73	*p=0. 002
Punishing	1.75±0.76	2.12±0.84	*p=0.001
Total McGill	8.3±2.35	9.16±2.14	*p=0.018

According to the results of the Visual Analogue Scale, there was not a meaningful relationship between the two groups in terms of pain level at 3-4 cm cervical dilatation and the second stage of childbirth. However, at the transitional stage (8-10 cm) pain level among the trained mothers was meaningfully less (Table 2).

The mean of pain scores using McGill scale during the active stage of delivery in both groups were compared. The results showed that in two subclasses from 4 subclasses, the total score of McGill scale was significantly less in the trained group (Table 3).

Both groups were matched with socio-demographic but between the two groups in terms of maternal education level, there was statistically significant difference. The effects of mother’s education level on the dependent variables (pain and anxiety) adjust with statistical analysis (Table 4).

**Table 4:** The effect of mother education in pain and anxiety in two studied groups

Mother Education	Pain intensity with VAS scale		Pain intensity with McGill scale		Anxiety	
	Control Group (mean±SD)	Classes Education (mean±SD)	Control Group (mean±SD)	Classes Education (mean±SD)	Control Group (mean±SD)	Classes Education (mean±SD)
Primary	9.46±16.8	79.23±22.15	9.37±2.01	0.5±2.44	16.69±5.09	15±5
High School	9.5±14.25	87.8±17.9	15±2.23	8.53±2.08	16.47±4.62	14.11±4.44
University	9.99±14.72	85.8±18.7	16±2.14	8.43±2.69	16.76±4.86	14.64±5.32
P-value	P=0.85	P=0.35	P=0.052	P=0.9	P=0.67	P=0.85

## 4. Discussion

According to the results of present study, prenatal education and the presence of a doula during labor and childbirth will significantly reduce childbirth stress and labor, in that trained women felt less childbirth stress and labor pain in comparison to the control group.

Clinical settings are stressful since they are full of unknowns and unexpected events and any disorder in physical functioning can cause anxiety (Smeltzers et al., 2008). For the majority of women, childbirth is a stressful event (Ceung & Ip, 2007). The stress during childbirth by increasing the stress inducing hormones like adrenaline and noradrenaline (Parsons, 2004; Engebretson, & Mahoney, 2004; Rosen, 2004). Increases the level of pain (Pilevarzadeh, Salari, & Shafiei, 2002; Mohamed et al., 2012), and reduce pleasant childbirth (Ceung & Ip, 2007).

Hosseini, Taghavi (2010), Ip et al. (2009) in their study showed that perinatal education can lessen the level of stress and labor pain, which is in line with our findings. Helen et al. (2005) conducted a research on 1197 mothers and studied the effects of perinatal education on childbirth and motherhood. Most of these mothers considered the education were helpful in controlling childbirth. Also, Husseininasab citing Robert wrote that by perinatal education, mothers learned how to solve their problems and define childbirth as a solvable problem.

American Local Midwife Association demonstrated the efficacy of perinatal education and justified it in that having knowledge in advance and the accompaniment of a trustworthy acquaintance are imperative when one is entering a strange, fearsome environment (Davis, 2007). Because entering labor room with unfamiliar personnel and plenty of measures and stages of a natural delivery are associated with may be stressful (Campell, Lake, & Falk, 2006). As this association suggests, perinatal education causes more positive attitudes toward childbirth and delivery room personnel, and increases their self-confidence (Davis, 2007). This was notably important in this study that each mother trusted their doulas, developed emotional interests to them, recognized them reliable, and felt less worried and more secure during delivery. Furthermore, these mothers developed better relationships with the delivery room personnel and collaborated more with them. Childbirth education program can promote the women’ scoping behavior and self-efficacy for childbirth (Hosseininasab & Taghavi, 2010; Ip, Tang & Goggins, 2009).

The findings of Gunasheela and Biliangady (2004) and Aajh et al. (2011) indicated that mothers participated in preparation classes developed better relationships with delivery room personnel. Feeling not prepared for giving birth, lack of confidence to delivery personnel play critical roles in increasing labor pain (Lowe, 1996).

In the present study, the McGill scale was employed to assess labor pain; the trained group meaningfully felt less pain than did the control group. Probably this was due to the changes in their viewpoint and making positive attitude to labor that the educated group seen labor less fearsome, less brutal and not as a punishment. Being Well informed during labor and childbirth can make mothers feel comfortable and calm and recognize the source of pain well. Therefore, they look at childbirth as a non-threatening life experience and collaborate and attempt to develop natural delivery (Lowe, 2002). The findings of Melzak et al. (1981) showed that preparation classes for childbirth, especially in cases that a good expert trains mothers, could emotionally reduce labor pain by 30% with the McGill Scale. That complies with our own findings. The Visual Analogue Scale that was employed to assess labor pain at the transitional stage of birth demonstrated less pain in trained group than by the control group. At the transitional stage that mothers feel severe pain, the accompaniment of a midwife and employment of non-medical comfort measures like massages, hot- and cold-water bags, sitting on a childbirth ball, and physiological breathing are useful. Melzak et al. (1975) demonstrated that if mothers going through natural delivery after preparation for it, they will experience less pain (by 30%) than unprepared ones according to Visual Analogue Scale of pain. Therefore, the best time for learning comfort measures is during pregnancy. Hodnett et al. (2002) concluded that mothers who were constantly supported during delivery and enjoyed comfort measures had shorter and less labor pain and took less pain killer. The studies in Iran also by Noori (2008), Rafiei (2012) and Samizadeh (2011) suggest being accompanied is effective because it alleviates labor pain (Javadnoori, Afshari, Montazeri, & Latifim, 2008; Vardanjani et al., 2012; Samieizadeh Toosi et al., 2011)

The severity of pain at stage two of childbirth showed no meaningful difference between the two groups. In fact, pain severity at stage one of childbirth can be under the influence of certain factors like fear, self-confidence, and believing in the ability todeliver, but certain interventions can be helpful for the parturient; however, their effect has not yet been confirmed for stage two (Vardanjani et al., 2012). The findings of this study are in agreement with the results found by Mohammaditabar et al. (2012) and Gognon et al. (1997). This does not comply with the findings of Pascali-Banaro and Roeger (2004). Mohammaditabar et al. (2012) showed that preparing mothers prior to delivery can meaningfully reduce the intensity of pain felt at stage one but it did not have any meaningful effect on the intensity of pain at stage two. The study setting employed gynecology beds in lithotomy position for delivery by hospital personnel. Therefore, doulas could not help to delivery. The second stage of delivery is a critical stage and the mother was surrounded by more of the personnel, but it was not possible for the doulas to give their psychological and physical support by comforting measures, so the lack of difference in the intensity of pain in the second stage between the two groups can be attributed to this restriction.

One of the restrictions of this study was that the cases were not selected randomly because we could not deprive a woman of attending training classes if she was interested. The subjects of the two groups were not matched for education; mothers having completed higher levels of education more likely took part in these classes. Then, the effect of the agent on the main variables was excluded in analysis.

## 5. Conclusion

The results of this study proved the efficacy of perinatal education. Moreover, it proved that supporting mothers reduces labor pain and agony during childbirth. It is imperative that during pregnancy all the issues relating to pregnancy and delivery, and the means of managing labor pain must be taught. This will help mothers actively control childbirth, overcome their fears and anxiety, and experience a pleasant delivery.
